# Disparities in antenatal care utilization and stillbirth risk among women of other origin than high‐income Western countries in Stockholm 2000–2020: A retrospective cohort study

**DOI:** 10.1111/aogs.70310

**Published:** 2026-07-20

**Authors:** Minna Lundén, Ingela Hulthén Varli, Helena Kopp Kallner, Hanna Åmark

**Affiliations:** ^1^ Department of Clinical Sciences, Danderyd Hospital Karolinska Institutet Stockholm Sweden; ^2^ BB S:t Göran Capio S:t Göran Hospital Stockholm Sweden; ^3^ Department of Women's and Children's Health Karolinska Institutet Stockholm Sweden; ^4^ Department of Women's Health, Division of Pregnancy and Childbirth Karolinska University Hospital Stockholm Sweden; ^5^ Department of Obstetrics and Gynecology Danderyd Hospital Stockholm Sweden; ^6^ Department of Clinical Science and Education, Unit of Obstetrics and Gynecology, Södersjukhuset Karolinska Institutet Stockholm Sweden; ^7^ Department of Obstetrics and Gynecology Södersjukhuset Stockholm Sweden

**Keywords:** epidemiology, obstetrics, stillbirth, Sweden

## Abstract

**Introduction:**

Foreign‐born women face a higher risk of adverse pregnancy outcomes, including stillbirth, compared to Swedish‐born women, and this disparity cannot be fully explained by known risk factors. This study investigates differences in pregnancy‐related health care by maternal origin and whether such differences may contribute to disparities in stillbirth risk.

**Material and Methods:**

This was a retrospective cohort study including all singleton births in the Stockholm region between 2000 and 2020 (*n* = 518 792 births). Data from the National Medical Birth Register was linked to data from Statistics Sweden and the VAL databases using the personal identity number of the mother. Maternal and fetal characteristics, inpatient care occasions, length of hospital stay, outpatient visits, and antenatal care visits were compared between women divided by maternal origin, experiencing live births, and stillbirths. Regression models using a forward selection strategy were performed, adjusting for different potential mediators on the causal pathway from maternal country of origin to stillbirth.

**Results:**

Among women with live births, those originating from Middle East/Northern Africa, sub‐Saharan Africa, and South America had significantly higher proportions of inpatient care occasions than women originating from high‐income Western Countries. Women with live births originating from other regions than high‐income Western Countries had significantly less antenatal care visits, regardless of parity. Among women with stillbirths, women originating from sub‐Saharan Africa had significantly more inpatient care occasions and fewer antenatal care visits than women originating from high‐income Western Countries. After adjusting for maternal risk factors, SGA infants, socioeconomic factors, multiple inpatient care occasions, and few antenatal care visits during pregnancy, women from sub‐Saharan Africa had a slightly elevated risk of stillbirth (aOR 1.29, 95% CI 1.00–1.66, *p*‐value 0.047) compared to women from high‐income Western Countries.

**Conclusions:**

Women originating from other regions than high‐income Western Countries, especially those from sub‐Saharan Africa, attended fewer antenatal care visits and had more inpatient care occasions compared to women from high‐income Western Countries. Reduced attendance in antenatal care could potentially be associated with their elevated stillbirth risk. Improving access to and participation in antenatal care should be prioritized to reduce disparities in stillbirth risk.

## Graphical abstract

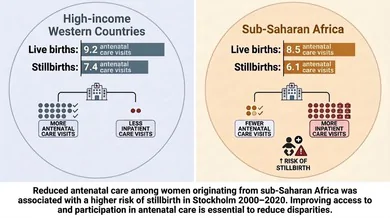

**Abbreviations**
BMI: body mass index
GA: gestational age
LGA: large for gestational age
OR: odds ratio
SD: standard deviation
SGA: small for gestational age

**Key Message**

Women originating from other regions than high‐income Western Countries had fewer visits in antenatal care during pregnancy. Reduced attendance in antenatal care may be a contributor to the elevated stillbirth risk observed among women originating from sub‐Saharan Africa.

## 1 INTRODUCTION

Adverse pregnancy and birth outcomes have been consistently reported, both nationally and internationally, to occur more frequently among foreign‐born women.^1^, ^2^, ^3^, ^4^, ^5^, ^6^ These disparities are thought to stem from a complex interplay of different factors including genetic predispositions, physical and social environments, and inequities in access to and quality of health care.^7^ Furthermore, racial discrimination has been identified as a significant risk factor for adverse birth outcomes.^8^

The utilization of antenatal health care plays a crucial role in identifying women at higher risk of stillbirth and ensuring appropriate monitoring and interventions. A Cochrane overview of systematic reviews found that midwife‐led models of care, particularly in settings where midwives act as primary health care providers for low‐risk pregnancies, were associated with reduced rates of stillbirth and perinatal death.^9^ Previous studies have also indicated that underutilization of antenatal care may increase the risk of stillbirth, particularly among foreign‐born women.^9^, ^10^, ^11^

In a previous study from our research group, we demonstrated that women in Sweden with origin from sub‐Saharan Africa face a significantly higher risk of stillbirth even after adjusting for known risk factors. Their elevated risk was partly mediated by giving birth to infants small for gestational age (SGA) and by socioeconomic factors, yet it could not be fully explained.^12^ We hypothesize that differences in patterns of health care utilization may contribute to the remaining unexplained higher risk.

In Sweden, an earlier study has investigated antenatal care visits among foreign‐born women in Malmö, showing an underutilization of planned antenatal visits but a higher frequency of unplanned visits to the delivery ward.^10^ However, no studies have explored if the frequency of visits differs among women of different maternal origin, who experience stillbirth or not. Therefore, the aim of this study was to examine potential differences in both the frequency and type of health care visits during pregnancy among women with different maternal origins, experiencing stillbirth or not.

## 2 MATERIAL AND METHODS

This was a retrospective cohort study of all singleton births in the Stockholm region between 2000 and 2020. The cohort was defined as the women who had an ICD code O80–O83 (Singleton deliveries) or O364 (Maternal care for intrauterine death) according to ICD‐10^13^ in the VAL‐databases. The VAL‐databases are administrative health care databases managed by the Centre for Health Data in the Stockholm Region. They contain anonymized data on all individuals' contacts with health care that receive compensation from the region, both regarding in‐ and outpatient care. The cohort was linked to the National Medical Birth Register and Statistics Sweden to obtain background characteristics. The National Medical Birth Register is a register provided by the Swedish government agency The National Board of Health and Welfare. Since 1973, it has recorded information from antenatal, obstetric and neonatal records in Sweden. It includes approximately 98% of births in Sweden with high quality due to partly automated data extraction from standardized regional electronic health care records.^14^ From Statistics Sweden we obtained maternal country of origin, socioeconomic factors and length of stay in days in Sweden for foreign‐born women. After excluding births with lethal malformations, 518 792 singleton births were included. As some women had more than one birth during the studied time period, the cohort included 313 756 unique women.

Records of all health care contacts occurring within 6 months prior to the delivery date were obtained from the VAL‐databases. These included all inpatient admissions and outpatient care visits. Multiple inpatient care occasions were defined as one or more inpatient admissions during pregnancy for reasons other than delivery. Outpatient care visits were defined as all visits conducted in outpatient care settings, regardless of health care facility.

In Sweden, all women are offered free of charge antenatal care throughout pregnancy, birth and the puerperium. Midwives provide most of the care given during pregnancy and childbirth, using standardized charts to record information during the perinatal and postnatal period. If needed, the midwife consults an obstetrician or a general practitioner.^15^ At the first antenatal care visit, a risk assessment is made and accordingly, a program for visits during the pregnancy is suggested. Many women exhibit one or more risk factors that imply care outside the standard program, up to 78% in the latest report from The National Pregnancy Register.^16^ There is no current national recommendation for the number of antenatal care visits in the standard program, leading to regional differences, but usually 9–11 visits are recommended.^17^ In 2024, the mean number of visits per pregnancy in Sweden was nine.^16^ The number of antenatal care visits during pregnancy in this study was obtained from The National Medical Birth Register.

Pregnancies requiring additional monitoring and follow‐up are referred to specialist antenatal care at the delivery unit for planning and follow‐up, while routine antenatal care visits continue in parallel. The visits at the specialist antenatal care were included among the outpatient care visits in this study.

From the National Medical Birth Register we obtained data on stillbirth, maternal age, body mass index (BMI), gestational age (GA) at birth, induction of labor, smoking status, invasive prenatal testing, assisted reproductive technology, parity, previous stillbirth, maternal medical diagnoses, and birthweight.

Stillbirth was defined as antepartum or intrapartum fetal death where the infant was born without signs of life from gestational week 22 + 0 according to the World Health Organization and ICD‐10 definition.^13^, ^18^ Maternal age was defined as the age in years at the time of birth. BMI was calculated using self‐reported or measured height and measured weight at the first recorded visit in antenatal care. According to the World Health Organization definition,^19^ underweight was defined as BMI less than 18.5, normal weight as BMI 18.5–24.9, overweight as BMI 25–29.9, and obesity as BMI equal to or over 30.

GA at birth was based on dating from the routinely offered mid‐trimester fetal ultrasound scan performed in gestational week 18–20 or the earlier ultrasound for combined ultrasound and biochemistry testing in gestational week 11 + 0–13 + 6, if the biparietal diameter was over 21 mm. If an ultrasound had not been performed during pregnancy, GA was calculated from the first day of the last menstrual period as stated in the electronic patient record. GA was categorized into: < 28 + 0 weeks, 28 + 0–31 + 6 weeks, 32 + 0–36 + 6 weeks, 37 + 0–40 + 6 weeks and≥ 41 + 0 weeks. Induction of labor was defined as a delivery starting by induction.

Maternal smoking was defined as self‐reported smoking at the first visit in antenatal care. Invasive prenatal testing was defined as amniocentesis and/or chorionic villus sampling during pregnancy. Assisted reproductive technology was defined as in vitro fertilization leading to the current pregnancy. Parity and previous stillbirth were based on self‐reported information at the first recorded visit in antenatal care. Parity was categorized as either primiparous or multiparous.

The maternal medical diagnoses were obtained through ICD codes recorded at hospital discharge after delivery according to ICD‐10^13^: Diabetes mellitus type 1 (ICD code E10 or O240), Diabetes mellitus type 2 (ICD code E11 or O241), gestational diabetes mellitus (ICD code O244), preeclampsia (ICD code O14), essential hypertension (ICD code I10–I15 or O10) and gestational hypertension (ICD code O13).

According to international standard, SGA was defined as birthweight less than the 10th percentile from the gender‐specific mean weight for the GA.^20^, ^21^ Similarly, large for gestational age (LGA) was defined as birthweight above the 90th percentile from the gender‐specific mean weight for the GA.^20^, ^21^

From Statistics Sweden, we obtained maternal country of origin, highest attained educational level, and disposable income per consumption unit. Maternal country of origin was categorized into six areas of origin: high‐income Western Countries (Sweden, the Nordic Countries, Western Europe, the United States, Canada, New Zealand, and Australia), Eastern Europe, Middle East/Northern Africa, sub‐Saharan Africa, Asia, and South America. Disposable income per consumption unit is a weight system used by Statistics Sweden in which the household's economic standard is calculated by dividing the sum of all family members' disposable income by the consumption weight that applies for the household. We divided the disposable income per consumption unit into quartiles for each respective study year to adjust for economic growth in society, with the lowest income quartile defined as one and the highest income quartile as 4. The highest attained educational level in the year of the current pregnancy was defined as primary school (up to 9 years of school), secondary school (10–12 years of school), and tertiary education.

### 2.1 Statistical methods

Differences in maternal and fetal characteristics were analyzed among women divided into groups based on maternal country of origin. Multiple inpatient care occasions and subsequent length of stay at the hospital, number of outpatient care visits, and number of antenatal care visits were compared between women with live births and stillbirths, divided into groups based on maternal country of origin. Data were presented as mean and standard deviation (SD) for continuous variables and as numbers and proportions for categorical variables. Continuous variables were compared using Welch *t*‐test, and categorical variables using Chi‐squared test.

The number of antenatal care visits was further compared between women with live births and stillbirths, divided into groups based on maternal country of origin and parity, as primiparous women usually are recommended more visits during pregnancy than multiparous women. The comparisons were made with Welch *t*‐test and presented as mean and SD. We also performed a sub analysis on the number of antenatal care visits according to GA at birth, as the number of visits naturally increases with longer pregnancies. We used a linear regression model to estimate the adjusted mean number of antenatal care visits for each maternal country of origin, accounting for parity, and GA category. Pairwise comparisons between maternal country of origin and high‐income Western Countries were obtained from estimated marginal means.

For all analyses above, Bonferroni correction was used to adjust the significance level based on the number of tests, as we did multiple comparisons in each group.

The number of antenatal care visits was analyzed in relation to length of residence in Sweden among foreign‐born women. The distribution of visits across categories of length of residence was illustrated using a boxplot. Differences in the mean number of visits were assessed using one‐way ANOVA. Post hoc pairwise comparisons were performed using Tukey's honestly significant difference (HSD) test to identify which groups differed.

Logistic regression analyses were made with a forward selection strategy adjusting for potential mediators on the causal pathway from maternal country of origin to stillbirth. As some women had more than one birth during the studied time period, we used a generalized estimating equation to adjust for potential within‐individual correlation. After performing a crude logistic regression analysis with stillbirth as outcome and maternal country of origin as exposure, we adjusted for age, parity, BMI, previous stillbirth, assisted reproductive therapy, and smoking. In a second adjusted analysis, we also adjusted for six maternal medical diagnoses (type I diabetes mellitus, type II diabetes mellitus, gestational diabetes mellitus, chronic hypertension, gestational hypertension, and preeclampsia) and the socioeconomic variables: educational level and disposable income per consumption unit. In the third analysis, we adjusted for the above mediators and for multiple care occasions, and in the final analysis, we also adjusted for few antenatal care visits, defined as < 7 visits during pregnancy. GA was considered a collider on the causal pathway from maternal country of origin to stillbirth and was not adjusted for. We did not adjust for outpatient care visits as they cover all visit types regardless of health care setting, and no clear threshold for a standard number of visits could be established.

Among the mediators studied, 14.3% of the included births had missing values on one or more mediators. BMI was the mediator missing the most, in 9.0% of births. Missing data were handled by using multiple imputation by chained equations (MICE) with 10 datasets. Analyses were performed on each imputed dataset and combined using Rubin's rules. As a sensitivity analysis, a complete case analysis was also performed.

The frequency of births among women of different maternal origins was compared, divided into three time periods: 2000–2006, 2007–2013, and 2014–2020.

The most common primary diagnoses for outpatient visits and multiple inpatient care occasions were compared among women with live births and women with stillbirths. The groups were not divided according to country of origin as the number of diagnoses among women with stillbirths in some groups was very few. Specific diagnoses from antenatal care visits were not available for analysis as the number of antenatal care visits was obtained from the National Medical Birth Register, which does not contain diagnoses for individual visits.

All statistical analyses were made with the statistical software R cran version 4.2.1 (https://cran.r‐project.org/).

## 3 RESULTS

The stillbirth incidence in the study was 0.26% (1333 stillbirths out of 518 792 births). The stillbirth incidence among women from sub‐Saharan Africa was higher than among women originating from high‐income Western Countries (0.64% compared to 0.22%) (Table 1).

**TABLE 1:** Maternal and fetal characteristics of women with singleton births in the Stockholm Region 2000–2020 divided into groups of maternal country of origin.

	High‐income Western Countries	Eastern Europe	Middle East/Northern Africa	Sub‐Saharan Africa	Asia	South America
***n***	386 775	26 951	51 956	22 017	19 538	11 555
Age mean (SD)	31.72 (4.83)	30.89 (5.09)	30.20 (5.53)	31.03 (5.64)	31.59 (4.96)	31.69 (5.46)
Age > 35 years	109 336 (28.3)	6550 (24.3)	12 103 (23.1)	6005 (27.3)	5580 (28.6)	3697 (32.0)
**BMI *n* (%)**						
Underweight	9056 (2.6)	980 (3.9)	1052 (2.2)	872 (4.3)	1217 (6.8)	166 (1.6)
Normal weight	238 850 (68.9)	16 821 (66.9)	24 520 (51.2)	9169 (45.4)	12 101 (67.5)	5652 (53.9)
Overweight	70 617 (20.4)	5300 (21.1)	15 276 (31.9)	6268 (31.0)	3535 (19.7)	3073 (29.3)
Obese	28 193 (8.1)	2038 (8.1)	7000 (14.6)	3882 (19.2)	1072 (6.0)	1588 (15.2)
**GA at birth *n* (%)**						
< 28 + 0	878 (0.2)	81 (0.3)	177 (0.3)	132 (0.6)	76 (0.4)	46 (0.4)
28 + 0–31 + 6	1438 (0.4)	86 (0.3)	209 (0.4)	151 (0.7)	79 (0.4)	56 (0.5)
32 + 0–36 + 6	12 754 (3.3)	820 (3.1)	1677 (3.2)	633 (2.9)	960 (4.9)	460 (4.0)
37 + 0–40 + 6	296 099 (76.9)	20 791 (77.4)	42 103 (81.4)	15 470 (70.5)	15 930 (82.0)	9391 (81.6)
≥ 41 + 0	74 073 (19.2)	5076 (18.9)	7581 (14.7)	5546 (25.3)	2390 (12.3)	1553 (13.5)
Induction of labour *n* (%)	60 138 (15.5)	4153 (15.4)	7670 (14.8)	4964 (22.5)	2802 (14.3)	1912 (16.5)
Smoker *n* (%)	16 994 (4.7)	2117 (8.1)	2335 (4.7)	379 (1.8)	471 (2.5)	347 (3.2)
Invasive prenatal testing *n* (%)	18 359 (4.7)	882 (3.3)	1349 (2.6)	485 (2.2)	679 (3.5)	548 (4.7)
Assisted reproductive therapy *n* (%)	16 556 (4.3)	1063 (3.9)	1382 (2.7)	279 (1.3)	753 (3.9)	358 (3.1)
Primiparous *n* (%)	185 737 (48.0)	13 192 (48.9)	19 694 (37.9)	6759 (30.7)	9550 (48.9)	4849 (42.0)
Previous stillbirth *n* (%)	1980 (0.5)	155 (0.6)	604 (1.2)	413 (1.9)	137 (0.7)	76 (0.7)
Diabetes mellitus type 1 *n* (%)	2022 (0.5)	46 (0.2)	79 (0.2)	57 (0.3)	28 (0.1)	20 (0.2)
Diabetes mellitus type 2 *n* (%)	169 (0.0)	19 (0.1)	121 (0.2)	106 (0.5)	86 (0.4)	20 (0.2)
Gestational diabetes mellitus *n* (%)	2974 (0.8)	424 (1.6)	1354 (2.6)	773 (3.5)	697 (3.6)	197 (1.7)
Preeclampsia *n* (%)	10 656 (2.8)	548 (2.0)	946 (1.8)	741 (3.4)	465 (2.4)	313 (2.7)
Chronic hypertension *n* (%)	2002 (0.5)	164 (0.6)	134 (0.3)	111 (0.5)	83 (0.4)	42 (0.4)
Gestational hypertension *n* (%)	7790 (2.0)	471 (1.7)	416 (0.8)	256 (1.2)	244 (1.2)	120 (1.0)
SGA *n* (%)	30 610 (7.9)	2463 (9.1)	6881 (13.3)	3303 (15.0)	2750 (14.1)	1082 (9.4)
LGA *n* (%)	38 409 (9.9)	2138 (7.9)	3357 (6.5)	1353 (6.2)	1325 (6.8)	1103 (9.6)
Stillbirth *n* (%)	862 (0.2)	64 (0.2)	177 (0.3)	141 (0.6)	58 (0.3)	31 (0.3)
Disposable income group^a^ mean (SD)	3.09 (1.05)	2.33 (1.18)	1.82 (1.05)	1.65 (0.93)	2.41 (1.21)	2.28 (1.15)
**Educational level *n* (%)**						
Primary	24 403 (6.4)	1495 (6.5)	10 917 (24.6)	6791 (36.4)	2508 (15.0)	1517 (14.3)
Secondary	124 139 (32.5)	6779 (29.7)	14 780 (33.3)	7444 (39.9)	4451 (26.6)	4056 (38.2)
Tertiary	233 900 (61.2)	14 589 (63.8)	18 634 (42.0)	4430 (23.7)	9763 (58.4)	5047 (47.5)

*Note*: Data are presented as mean and standard deviation (SD) for continuous variables and as numbers and proportions for categorical variables.

Abbreviations: BMI, body mass index; GA, gestational age; LGA, large for gestational age; SGA, small for gestational age.

^a^ Disposable income group mean: the disposable income per consumption unit divided into quartiles for each respective study year, with the lowest income quartile defined as 1 and the highest income quartile as 4.

Women originating from the Middle East/Northern Africa and sub‐Saharan Africa were more often multiparous and overweight/obese compared to women of other origin. Women originating from the Middle East/Northern Africa, sub‐Saharan Africa, and Asia more often gave birth to SGA infants and less often to LGA infants even though the incidence of gestational diabetes mellitus and type 2 diabetes mellitus, often associated with LGA, in these groups was higher (Table 1). Women from sub‐Saharan Africa more often reached 41 + 0 gestational weeks before giving birth and were induced to a higher extent (Table 1).

In the cohort, 92.3% (479 088 of 518 792) of the pregnant women had only one inpatient care occasion (i.e. delivery). The range of inpatient care occasions for the remaining 39 704 women was 2–20, in total 51 875 inpatient care occasions. Women with live births originating from the Middle East/Northern Africa, sub‐Saharan Africa, and South America had a significantly higher proportion of inpatient care occasions in comparison to women originating from high‐income Western Countries (8.8%, 9.9% and 11.0% compared to 7.3%) (Table 2). Among women with stillbirths, women from sub‐Saharan Africa had a significantly higher proportion of inpatient care occasions compared to women originating from high‐income Western Countries (24.1% compared to 14.0%) (Table 2). For the inpatient care occasions other than birth, women from sub‐Saharan Africa with live births had a longer mean period of care compared to women originating from high‐income Western Countries (3.66 days compared to 3.18 days) (Table 2).

**TABLE 2:** Comparison of health care utilization among women in the Stockholm region with singleton live births and stillbirths 2000–2020 divided into groups of maternal country of origin.

	High‐income Western Countries		Eastern Europe		Middle East/Northern Africa		Sub‐Saharan Africa		Asia		South America	
	Live births	Stillbirths	Live births	Stillbirths	Live births	Stillbirths	Live births	Stillbirths	Live births	Stillbirths	Live births	Stillbirths
***n***	385 913	862	26 887	64	51 779	177	21 876	141	19 480	58	11 524	31
Multiple inpatient care occasions *n* (%)^a^	28 208 (7.3)	121 (14.0)	1857 (6.9)^b^	9 (14.1)	4540 (8.8)^b^	27 (15.3)	2157 (9.9)^b^	34 (24.1)^c^	1472 (7.6)	13 (22.4)	1267 (11.0)^b^	4 (12.9)
Period of care days mean (SD)	3.18 (4.28)	3.52 (3.15)	3.15 (4.95)	4.42 (7.15)	3.14 (9.40)	5.93 (8.08)	3.66 (5.02)^b^	4.07 (4.80)	3.28 (4.78)	2.29 (1.31)	3.22 (3.75)	8.25 (6.18)
Visits in outpatient care mean (SD)	12.0 (9.8)	11.8 (12.3)	12.7 (8.7)^b^	11.8 (7.5)	12.5 (9.2)^b^	12.9 (14.3)	12.7 (9.5)^b^	12.1 (8.9)	12.0 (8.5)	12.1 (9.8)	12.7 (10.2)^b^	12.5 (8.9)
Antenatal care visits mean (SD)	9.2 (3.0)	7.4 (4.2)	8.6 (2.9)^b^	6.4 (4.1)	8.6 (3.1)^b^	7.4 (8.9)	8.5 (3.1)^b^	6.1 (3.8)^c^	8.5 (2.9)^b^	7.9 (4.4)	8.6 (3.1)^b^	7.2 (4.1)

*Note*: Data are presented as mean and standard deviation (SD) for continuous variables and as numbers and proportions for categorical variables.

^a^ Multiple inpatient care occasions: having one or more inpatient care occasion during pregnancy for other reasons than delivery.

^b^ Significant difference compared to women from high‐income Western Countries with live births.

^c^ Significant difference compared to women from high‐income Western Countries with stillbirths.

Women with live births originating from Eastern Europe, the Middle East/Northern Africa, sub‐Saharan Africa, and South America had significantly more outpatient visits than women with live births originating from high‐income Western Countries. Among women with stillbirths, there were no significant differences between the number of outpatient visits when comparing women of different maternal origin (Table 2).

Women with live births originating from other regions than high‐income Western Countries had significantly less antenatal care visits (Table 2), regardless of being primi‐ or multiparous (Table 3). Among women with stillbirths, the women originating from sub‐Saharan Africa had fewer antenatal care visits in comparison to women originating from high‐income Western Countries (Table 2). When divided based on parity, the difference was not statistically significant after correction for multiple comparisons (*p* = 0.027, Bonferroni‐adjusted *p =* 0.135 among primiparous women, and *p =* 0.018, Bonferroni‐adjusted *p =* 0.090 among multiparous women) (Table 3).

**TABLE 3:** Mean number of visits within antenatal care among women with singleton live‐ and stillbirths in the Stockholm region between 2000 and 2020 divided according to maternal region of origin and parity.

	High‐income Western Countries		Eastern Europe		Middle East/Northern Africa		Sub‐Saharan Africa		Asia		South America	
	Live birth	Stillbirth	Live birth	Stillbirth	Live birth	Stillbirth	Live birth	Stillbirth	Live birth	Stillbirth	Live birth	Stillbirth
Mean visits in antenatal care among primiparous women (SD)	9.6 (3.1)	7.7 (4.3)	9.0 (3.0)^a^	7.2 (4.3)	9.2 (3.1)^a^	7.8 (4.3)	9.2 (3.3)^a^	6.4 (3.5)	8.9 (2.9)^a^	7.7 (4.6)	9.1 (3.2)^a^	8 (2.9)
Mean visits in antenatal care among multiparous women (SD)	8.8 (2.9)	7.1 (4.2)	8.2 (2.7)^a^	5.4 (3.9)	8.3 (3.0)^a^	7.1 (3.5)	8.1 (3.0)^a^	5.9 (4.0)	8.1 (2.8)^a^	8.1 (4.2)	8.3 (2.9)^a^	6.4 (4.9)

^a^ Significant difference compared to women originating from high‐income Western Countries with live births.

When the number of antenatal care visits was divided according to gestational length, the difference in the number of visits was most apparent at gestational length > 37 + 0 (Table 4). At that gestational length, women with live births originating from all other regions than high‐income Western Countries had significantly less antenatal care visits compared to women originating from high‐income Western Countries, but no significant differences were observed among pregnancies with a gestational length of less than 31 + 6 weeks. For women with stillbirths, the only observed significant difference was in the number of antenatal care visits among multiparous women originating from sub‐Saharan Africa at gestational length > 37 + 0 weeks, who had significantly less visits compared to women with stillbirths from high‐income Western Countries (7.2 compared to 9.5) (Table 4).

**TABLE 4:** Mean number of visits within antenatal care among women with singleton live‐ and stillbirths in the Stockholm region between 2000 and 2020 divided according to maternal region of origin, parity, and gestational age.

		High‐income Western Countries		Eastern Europe		Middle East/Northern Africa		Sub‐Saharan Africa		Asia		South America	
		Live birth	Stillbirth	Live birth	Stillbirth	Live birth	Stillbirth	Live birth	Stillbirth	Live birth	Stillbirth	Live birth	Stillbirth
Mean visits in antenatal care among primiparous women (SE)	GA <196 days	3.3 (1.9)	3.4 (2.3)	3.0 (1.3)	3.0 (0.0)	3.7 (2.0)	2.9 (0.8)	3.0 (1.5)	4.4 (2.9)	3.6 (2.2)	3.9 (2.0)	3.0 (1.4)	5.5 (3.5)
	GA 196–223 days	4.9 (2.5)	4.7 (1.6)	4.5 (1.8)	2.0 (0.0)	5.0 (2.4)	4.6 (2.4)	5.0 (2.9)	5.3 (0.8)	4.6 (2.1)	8.0 (0.0)	4.4 (2.4)	8.5 (0.7)
	GA 224–258 days	7.3 (2.9)	7.2 (2.3)	7.0 (2.9)	9.2 (1.5)	6.9 (2.7)^a^	7.7 (2.9)	7.0 (3.3)	6.5 (4.0)	6.9 (2.3)^a^	6.7 (3.5)	6.9 (2.8)	6.0 (1.0)
	GA 259 days or more	9.8 (3.1)	10.3 (4.0)	9.1 (3.0)^a^	9.4 (4.0)	9.3 (3.1)^a^	10.1 (3.9)	9.3 (3.2)^a^	8.6 (3.5)	9.0 (2.9)^a^	10.8 (4.3)	9.2 (3.2)^a^	10.5 (2.5)
Mean visits in antenatal care among multiparous women (SE)	GA <196 days	3.5 (2.1)	3.1 (2.1)	2.8 (1.1)	3.1 (1.6)	3.2 (1.7)	4.1 (2.2)	3.5 (2.1)	3.6 (2.4)	3.3 (1.7)	4.0 (1.4)	2.3 (0.9)^a^	2.3 (0.6)
	GA 196–223 days	4.7 (2.5)	5.0 (2.5)	4.3 (1.8)	2.0 (0.0)	4.8 (2.7)	4.7 (2.7)	5.4 (4.7)	4.2 (2.4)	4.9 (2.2)	10.5 (2.1)	4.2 (1.8)	5.0 (0.0)
	GA 224–258 days	7.1 (3.2)	6.9 (3.5)	6.6 (2.9)^a^	6.0 (1.9)	6.6 (2.8)^a^	6.5 (3.3)	6.7 (3.3)	6.8 (5.4)	6.7 (3.0)	8.3 (6.1)	6.7 (3.0)	5.0 (4.2)
	GA 259 days or more	8.9 (2.9)	9.5 (3.9)	8.3 (2.7)^a^	9.7 (4.1)	8.4 (2.9)^a^	8.9 (3.1)	8.2 (2.9)^a^	7.2 (3.2)^b^	8.2 (2.8)^a^	9.6 (2.6)	8.4 (2.9)^a^	10.0 (5.6)

*Note*: GA <196 days corresponding to less than gestational week 28 + 0. GA 196–223 days corresponding to gestational week 28 + 0 to 31 + 6. GA 224–258 days corresponding to gestational week 32 + 0 to 36 + 6. GA 259 days or more corresponding to gestational week 37 + 0 and upwards.

Abbreviation: SE, standard error.

^a^ Significant difference compared to women originating from high‐income Western Countries with live births.

^b^ Significant difference compared to women originating from high‐income Western Countries with stillbirths.

**FIGURE 1:**
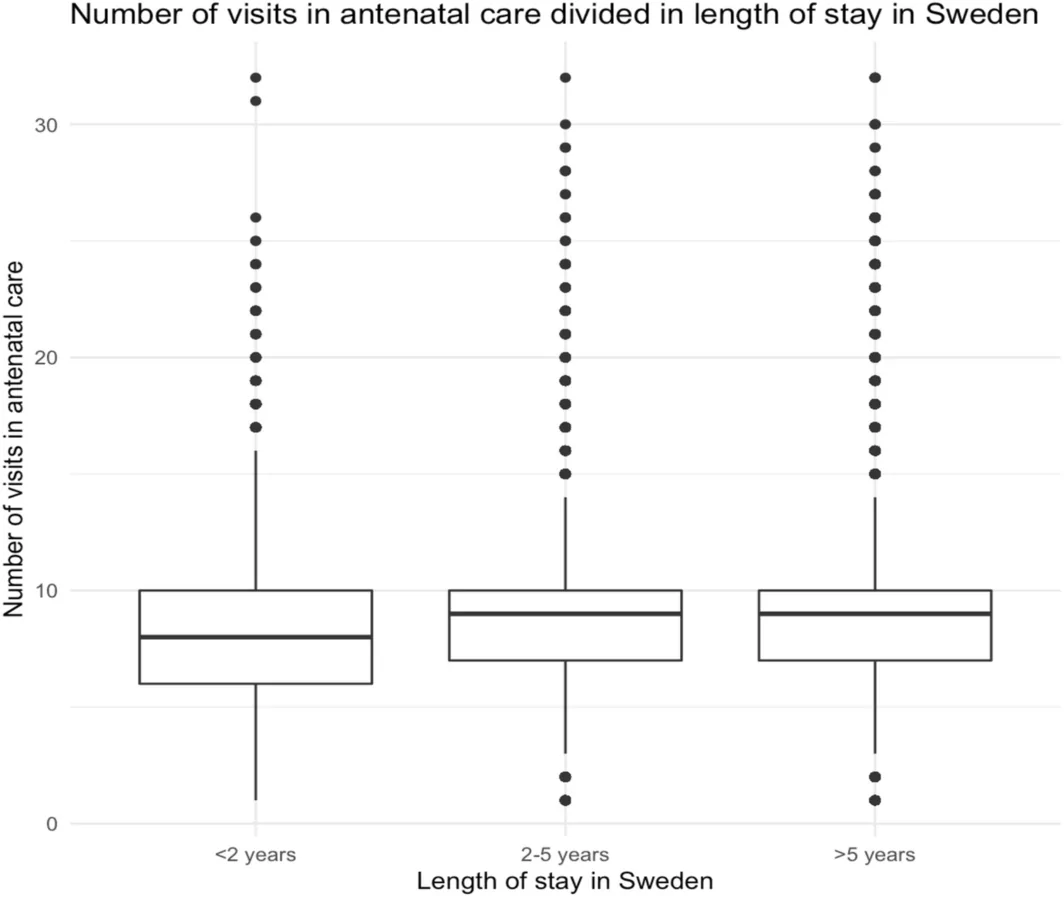
Number of antenatal care visits among pregnant women in Stockholm 2000–2020 originating from other regions than high‐income Western Countries divided in length of stay in Sweden.

Foreign‐born women who had been in Sweden < 2 years had significantly less antenatal care visits compared to foreign‐born women who had been in Sweden for 2 or more years. There was no significant difference in the number of antenatal care visits between foreign‐born women who had been in Sweden for 2–5 years compared to > 5 years (Figure 1).

In the crude logistic regression analysis, women originating from the Middle East/Northern Africa, sub‐Saharan Africa, and Asia had a significantly higher risk of stillbirth, with the highest risk observed among women originating from sub‐Saharan Africa (OR 2.93, 95% CI 2.44–3.51) (Table 5). After adjustment for maternal risk factors, SGA infants, and socioeconomic factors, the risk remained significantly elevated among women from sub‐Saharan Africa (aOR 1.43, 95% CI 1.12–1.82), whereas no significant differences were observed for the other groups compared to women originating from high‐income Western Countries. Additional adjustment for multiple inpatient care occasions had little impact on the estimate (aOR 1.41, 95% CI 1.10–1.81). However, further adjustment for fewer than seven antenatal care visits attenuated the association, rendering it only marginally significant (aOR 1.29, 95% CI 1.00–1.66) (Table 5). In the complete case analysis, the adjusted odds ratios differed slightly (Table S1).

**TABLE 5:** Logistic regression model with stillbirth as outcome and country of origin as exposure of all singleton births in the Stockholm Region 2000–2020.

	High‐income Western Countries	Eastern Europe			Middle East/Northern Africa			Sub‐Saharan Africa			Asia			South America		
	OR	OR	95% CI	*p*	OR	95% CI	*p*	OR	95% CI	*p*	OR	95% CI	*p*	OR	95% CI	*p*
Crude logistic regression	1.0 (ref)	1.07	0.82–1.39	0.6	1.55	1.32–1.83	< 0.001	2.93	2.44–3.51	< 0.001	1.34	1.02–1.76	0.036	1.19	0.81–1.72	0.4
Adjusted for age, parity, BMI, previous stillbirth, assisted reproductive therapy and smoking	1.0 (ref)	1.09	0.83–1.44	0.5	1.50	1.25–1.80	< 0.001	2.79	2.28–3.41	< 0.001	1.41	1.05–1.89	0.02	1.03	0.68–1.56	0.9
Adjusted for mediators above, fetuses small for gestational age, maternal medical diagnoses^a^, educational level and disposable income	1.0 (ref)	0.92	0.67–1.26	0.6	1.06	0.86–1.31	0.5	1.43	1.12–1.82	0.004	0.99	0.71–1.36	0.9	0.87	0.57–1.34	0.5
Adjusted for mediators above and multiple care occasions	1.0 (ref)	0.92	0.68–1.26	0.6	1.05	0.85–1.30	0.6	1.41	1.10–1.81	0.007	0.98	0.70–1.38	0.9	0.85	0.56–1.33	0.5
Adjusted for mediators above and less than 7 visits in antenatal care	1.0 (ref)	0.88	0.64–1.22	0.4	1.03	0.84–1.27	0.8	1.29	1.00–1.66	0.047	0.92	0.66–1.29	0.6	0.80	0.51–1.24	0.3

Abbreviation: BMI = body mass index.

^a^ Diabetes mellitus type I and II, gestational diabetes mellitus, chronic hypertension, gestational hypertension, and preeclampsia.

The mean number of births among women of different origin shows a decline in the number of births among women from high‐income Western Countries and women from South America, but a rise in the number of births among women from Eastern Europe, the Middle East/North Africa, sub‐Saharan Africa, and Asia (Table 6).

**TABLE 6:** Mean number of births/year in the Stockholm Region among women of different origin.

	2000–2006	2007–2013	2014–2020
High‐income Western Countries	18 286	21 390	20 818
Eastern Europe	720	1451	2027
Middle East/Northern Africa	2082	2842	3364
Sub‐Saharan Africa	811	1239	1525
Asia	685	1038	1349
South America	534	724	637

The most common primary diagnoses for inpatient care occasions other than birth and outpatient visits for women with live births and stillbirths are presented in the supplementary material (Tables S2 and S3). All inpatient care occasions had a primary diagnosis, but among the outpatient care visits, there was a high frequency of missing primary diagnoses, around 50%.

## 4 DISCUSSION

Women with live births from regions other than high‐income Western Countries had fewer antenatal care visits than women from high‐income Western Countries, regardless of parity. The difference was most pronounced in term pregnancy. For women originating from sub‐Saharan Africa, who reached gestational week ≥ 41 + 0 more often than the other studied groups of maternal origin, the number of antenatal care visits would be expected to be higher as the number of visits increases with increasing gestational length.

In contrast, women with live births originating from the Middle East/Northern Africa, sub‐Saharan Africa, and South America had a significantly higher proportion of inpatient care occasions compared with women originating from high‐income Western Countries. These findings are consistent with a previous study from Malmö, Sweden, which reported lower utilization of planned antenatal care among some groups of foreign‐born women.^10^ Instead, these women were more likely to make unplanned visits to the delivery ward. It was suggested that the delivery ward is not designed to manage routine antenatal care, meaning that some foreign‐born women may not fully benefit from the continuity and preventive aspects of planned antenatal care. In hospital settings, where women often meet different midwives and obstetricians, the lack of continuity of care may increase the risk of missing important parts of the medical history.

Among women with stillbirths originating from sub‐Saharan Africa, a higher frequency of inpatient care occasions and longer hospital stays was observed, while also having fewer antenatal care visits during pregnancy compared to women with stillbirths originating from high‐income Western Countries. Inpatient care occasions for other reasons than delivery may result from unplanned visits at the delivery ward due to self‐experienced symptoms or referrals from the antenatal care midwives. In this register‐based study, we cannot distinguish between these reasons, though they likely affect the results differently. Swedish maternity care is primarily organized around antenatal care visits, with access to an obstetrician or a general practitioner for consultation if complications arise, and referral to the delivery ward for more severe cases. Fewer antenatal care visits reduce opportunities for midwifes to detect complications, but in some cases, women may directly contact the delivery ward due to symptoms, potentially making antenatal visits less critical. However, many pregnancy complications—such as gestational hypertension, pre‐eclampsia and intrauterine growth restriction—may not present with overt symptoms yet significantly impact outcomes, including stillbirth.^22^, ^23^, ^24^ Detecting complications allows for closer monitoring and timely interventions, such as timing of delivery, which has contributed to the decreasing incidence of stillbirth among women with preeclampsia.^25^

Women of other origin than high‐income Western Countries who had lived in Sweden for less than 2 years had significantly less antenatal care visits compared to those who had lived in Sweden for a longer period. One explanation could be that a shorter duration of stay reduces the number of visits possible to attend. However, newly arrived women of other origin than high‐income Western Countries may face multiple barriers to accessing and understanding Swedish antenatal care, including language difficulties and limited knowledge of available services. Some may not fully understand the purpose of antenatal care, particularly if preventive care is less common in their country of origin and care is typically sought only when problems arise.^26^ Ensuring that clear, culturally sensitive information about free antenatal care and its role in early risk assessment and monitoring is effectively communicated could help facilitate timely engagement and more equitable care.

The excess risk of stillbirth among women from sub‐Saharan Africa decreased after adjustment for maternal and socioeconomic factors and was further reduced when adjusting for fewer than seven antenatal care visits. This finding suggests that adequate antenatal care mitigates part of the remaining excess risk. The explanation is probably a synergistic effect as the antenatal care visits might reveal risk factors, present before or emerging during pregnancy, and therefore increase the possibility to intervene in time. This has been highlighted earlier by the National Board of Health and Welfare^27^ and our results align with a previous study from New Zeeland, which found that having less than half of the recommended visits in antenatal care was associated with more than a twofold increase in the risk of stillbirth compared with attending the recommended number of visits.^28^ Multiple inpatient care occasions did not appear to substantially alter the risk of stillbirth in our study, but may influence the number of antenatal care visits, as time spent in the hospital might limit attendance.

An increase in the mean number of births was observed among women from Eastern Europe, the Middle East/Northern Africa, sub‐Saharan Africa, and Asia. This demographic trend underscores the importance of planning for future antenatal care capacity, ensuring that healthcare professionals have sufficient time and training to provide individualized and culturally sensitive support for all pregnant women, regardless of background or risk profile. This is in line with the recent recommendations from the National Board of Health and Welfare.^29^

A strength in this study is the possibility to link time spent in Sweden for women of other origin than high‐income Western Countries to antenatal care utilization, highlighting how duration of residence influences attendance to care. This raises important questions how newly arrived women of other origin than high‐income Western Countries are introduced to the Swedish antenatal care system. However, a limitation is the high frequency of outpatient visits without a recorded primary diagnosis, which restricts the ability to interpret reasons for seeking such care. Another limitation is that the available data did not allow for an assessment of the timing of the first visit in antenatal care or if an interpreter was used during visits in health care for women of other origin than high‐income Western Countries. Knowledge of these factors could have provided more understanding about the underlying mechanisms as late enrolment in antenatal care and language barriers are considered risk factors for adverse outcomes in pregnancy.

## 5 CONCLUSION

This study highlights important disparities in the utilization of antenatal care among women of other origin than high‐income Western Countries in Sweden. Women of other origin than high‐income Western Countries, particularly women originating from sub‐Saharan Africa, had fewer antenatal care visits and a higher proportion of inpatient care occasions compared to women from high‐income Western Countries. Adjustment for the number of antenatal care visits attenuated the elevated stillbirth risk among women from sub‐Saharan Africa, suggesting that reduced engagement in antenatal care could potentially be associated with adverse outcomes. Efforts to improve awareness, accessibility but also attendance to antenatal care–especially among recently arrived women of other origin than high‐income Western Countries–should therefore be prioritized to promote equitable maternal health and reduce the risk of stillbirth.

## AUTHOR CONTRIBUTIONS

All authors participated in the study design. Ingela Hulthén Varli, Hanna Åmark, and Minna Lundén applied for the ethical permit and amendments. Minna Lundén and Hanna Åmark retrieved data and performed data analysis. All authors participated in data interpretation. Minna Lundén drafted the initial version of the manuscript, which was revised by all authors who also approved the final manuscript.

## FUNDING INFORMATION

The authors have nothing report.

## CONFLICT OF INTEREST STATEMENT

The authors declare no conflict of interest.

## ETHICS STATEMENT

Ethical approval for this study was obtained from the Swedish Ethical Review Authority Dnr 2020–01855, approved the 24th of June 2020, with amendment Dnr 2021–03412, approved the 9th of July 2021, enabling linking of the National Medical Birth Register, Statistics Sweden and Centre for Health Data, and amendment 2022–03758‐02, enabling a longer study period including all births up until the application for register data was made, approved the 3rd of August 2022.

## Supporting information


**Table S1.** Complete case analysis with a logistic regression model with stillbirth as outcome and country of origin as exposure of all singelton births in the Stockholm Region 2000–2020.

**Table S2.** The most common primary diagnoses for inpatient care occasions other than birth and outpatient visits for women with live births and stillbirth.

**Table S3.** The most common primary diagnoses for outpatient visits for women with live births and stillbirth.

## Data Availability

The data that support the findings of this study are available on request from the corresponding author. The data are not publicly available due to privacy or ethical restrictions.
