# Supplementary material for: Disparities in antenatal care utilization and stillbirth risk among women of other origin than high‐income Western countries in Stockholm 2000–2020: A retrospective cohort study
Source: Acta Obstet Gynecol Scand. 2026 Jul 20;105(10):2030–40. doi: 10.1111/aogs.70310 (PMC13394951; doi:10.1111/aogs.70310)
Supplement: Supplementary file 1 [file AOGS-105-2030-s001.docx]

| **Live birth with inpatient care occasions other than birth (n=39496)** | | **Stillbirth with inpatient care occasions other than birth (n=208)** | |
| --- | --- | --- | --- |
| O470B | False labour before 37 completed weeks of gestation (n=4120) | O364 | Maternal care for intrauterine death (n=33) |
| O468 | Other antepartum haemorrhage (n=3218) | O470B | False labour before 37 completed weeks of gestation (n=17) |
| O471 | False labour at or after 37 completed weeks of gestation (n=2972) | O211 | Hyperemesis gravidarum with metabolic disturbance (n=16) |
| O268B | Other specified pregnancy-related conditions: abdominal pain (n=2933) | O468 | Other antepartum haemorrhage (n=10) |
| O470A | Preterm contractions without cervical maturation before 37 completed weeks of gestation (n=2875) | O200 | Threatened abortion (n=9) |

**Supplementary Table 2:** The most common primary diagnoses for inpatient care occasions other than birth and outpatient visits for women with live births and stillbirth.
