# Supplementary material for: Disparities in antenatal care utilization and stillbirth risk among women of other origin than high‐income Western countries in Stockholm 2000–2020: A retrospective cohort study
Source: Acta Obstet Gynecol Scand. 2026 Jul 20:10.1111/aogs.70310. Online ahead of print. doi: 10.1111/aogs.70310 (PMC13394951; doi:10.1111/aogs.70310)
Supplement: Supplementary file 1 — Table S1. Complete case analysis with a logistic regression model with stillbirth as outcome and country of origin as exposure of all singelton births in the Stockholm Region 2000–2020. [file AOGS-9999-0-s002.docx]

|  | High-income Western Countries | Eastern Europe | | | Middle East/  Northern Africa | | | Sub-Saharan Africa | | | Asia | | | South America | | |
| --- | --- | --- | --- | --- | --- | --- | --- | --- | --- | --- | --- | --- | --- | --- | --- | --- |
|  | OR | OR | 95 % CI | *p*-value | OR | 95 % CI | *p*-value | OR | 95 % CI | *p*-value | OR | 95 % CI | *p*-value | OR | 95 % CI | *p*-value |
| Crude logistic regression | 1.0 (ref) | 1.01 | 0.73-1.39 | 0.9 | 1.62 | 1.34-1.95 | <0.001 | 2.64 | 2.11-3.29 | <0.001 | 1.35 | 0.98-1.85 | 0.067 | 1.18 | 0.77-1.81 | 0.4 |
| Adjusted for age, parity, BMI, previous stillbirth, ART and smoking | 1.0 (ref) | 1.01 | 0.73-1.39 | 0.9 | 1.56 | 1.29-1.89 | <0.001 | 2.43 | 1.94-3.05 | <0.001 | 1.38 | 1.00-1.90 | 0.050 | 1.09 | 0.71-1.67 | 0.7 |
| Adjusted for mediators above and fetuses small for gestational age, maternal medical diagnoses*, educational level and disposable income | 1.0 (ref) | 0.92 | 0.66-1.26 | 0.6 | 1.05 | 0.85-1.28 | 0.7 | 1.38 | 1.08-1.77 | 0.011 | 1.00 | 0.72-1.38 | 1.0 | 0.88 | 0.58-1.36 | 0.6 |
| Adjusted for mediators above and multiple care occasions | 1.0 (ref) | 0.92 | 0.67-1.27 | 0.6 | 1.04 | 0.85-1.28 | 0.7 | 1.37 | 1.07-1.75 | 0.012 | 1.00 | 0.72-1.38 | 1.0 | 0.87 | 0.57-1.33 | 0.5 |
| Adjusted for mediators above and less than 7 visits in antenatal care | 1.0 (ref) | 0.88 | 0.64-1.21 | 0.4 | 1.01 | 0.83-1.24 | 0.9 | 1.25 | 0.98-1.60 | 0.073 | 0.93 | 0.67-1.29 | 0.7 | 0.80 | 0.52-1.22 | 0.3 |

**Supplemental table 1**. Complete case analysis with a logistic regression model with stillbirth as outcome and country of origin as exposure of all singelton births in the Stockholm Region 2000–2020.

* Diabetes mellitus type I and II, gestational diabetes mellitus, chronic hypertension, gestational hypertension and preeclampsia.
