# Supplementary material for: Disparities in antenatal care utilization and stillbirth risk among women of other origin than high‐income Western countries in Stockholm 2000–2020: A retrospective cohort study
Source: Acta Obstet Gynecol Scand. 2026 Jul 20:10.1111/aogs.70310. Online ahead of print. doi: 10.1111/aogs.70310 (PMC13394951; doi:10.1111/aogs.70310)
Supplement: Supplementary file 3 — Table S3. The most common primary diagnoses for outpatient visits for women with live births and stillbirth. [file AOGS-9999-0-s001.docx]

**Supplementary Table 3:** The most common primary diagnoses for outpatient visits for women with live births and stillbirth.

| **Live birth**  **(number of outpatient visits: 5977983)** | | **Stillbirth**  **(number of outpatient visits: 15070)** | |
| --- | --- | --- | --- |
| Z30-Z39 | Persons encountering health services in circumstances related to reproduction (n=528274) | Z30-Z39 | Persons encountering health services in circumstances related to reproduction (n=1554) |
| O268 | Other specified pregnancy-related conditions (n=201336) | O268 | Other specified pregnancy-related conditions (n=440) |
| Z80-Z99 | Persons with potential health hazards related to family and personal history and certain conditions influencing health status (n=108385) | O364 | Maternal care for intrauterine death (n=257) |
| O267 | Subluxation of symphysis (pubis) in pregnancy, childbirth and the puerperium (n=103746) | F40-F48 | Neurotic, stress-related and somatoform disorders (n=172) |
| O269 | Pregnancy-related condition, unspecified (n=93070) | O269 | Pregnancy-related condition, unspecified (n=154) |
